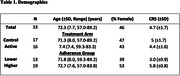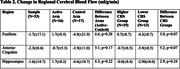# Precision Recommendations to Optimize Neurocognition [PREVENTION] Trial – Intervention Adherence Impacts Cerebral Blood Flow

**DOI:** 10.1002/alz.092449

**Published:** 2025-01-09

**Authors:** Jennifer E. Bramen, Prabha Siddarth, Emily S. Popa, John F. Hodes, Molly K. Rapozo, Ryan M. Glatt, Aarthi S. Ganapathi, Claudia L. Wong, Verna R. Porter, Mihae Kim, Gavin T Kress, William Sparks, Ynez M. Tongson, Stella E. Panos, Lee Hood, Jared C. Roach, David A. Merrill

**Affiliations:** ^1^ Saint John’s Cancer Institute at Providence Saint John’s Health Center, Santa Monica, CA USA; ^2^ Pacific Brain Health Center, Pacific Neuroscience Institute and Foundation, Santa Monica, CA USA; ^3^ David Geffen School of Medicine at University of California Los Angeles, Los Angeles, CA USA; ^4^ Drexel University College of Medicine, Philadelphia, PA USA; ^5^ Pacific Brain Health Center, Pacific Neuroscience Institute Foundation, Santa Monica, CA USA; ^6^ University of Southern California Department of Psychology, Los Angeles, CA USA; ^7^ Providence Saint John’s Health Center, Santa Monica, CA USA; ^8^ The Icahn School of Medicine at Mount Sinai, New York, NY USA; ^9^ Neuroscience Program, Union College, Schenectady, NY USA; ^10^ Institute for Systems Biology, Seattle, WA USA

## Abstract

**Background:**

Medical management and lifestyle are potentially crucial interventions for Alzheimer’s disease (AD). In this study, we present the relationship between adherence of a personalized multi‐modal intervention for AD on change in cerebral blood flow (CBF) after 12‐months.

**Methods:**

The PREVENTION study is an ongoing randomized clinical trial (McEwen, 2001). Thirty‐three participants with biomarker evidence of amyloidosis had completed the study at the time of the analysis (**Table 1**). While both arms received personalized multi‐modal lifestyle recommendations and four medical visits, the active arm also received dietary counseling, group physical and cognitive exercise, health coaching, and nutritional supplements free of charge. We examined the effects of the 1) the intervention and 2) adherence on CBF. We hypothesized that 1) the active arm and 2) higher intervention adherence would have improved CBF in regions related to level of physical activity (Kleinloog, 2019; Chapman, 2013) and those pertinent to AD. CBF was assessed using arterial spin labeling (ASL). Adherence was measured using the clinician rating scale (CRS), which uses a scale of 1‐7 (Kemp, 1998). Participants were divided into two groups based on a cutoff of 5 (passive acceptance). One participant was excluded from this analysis due to missing CRS data. Effects were assessed using a two‐tailed t‐test.

**Results:**

Treatment arms did not differ in any demographic measures at baseline or CRS. Preliminary findings indicate that regional blood flow declined over one year across the whole sample (**Table 2**). However, individuals with higher adherence experienced increased blood flow in the fusiform gyrus and less blood flow reduction, compared to those with lower adherence, in the anterior cingulate and hippocampus. Findings were borderline significant in the fusiform and anterior cingulate, but not the hippocampus.

**Conclusions:**

In this small sample, we found evidence that higher adherence increased or attenuated decline in CBF in regions impacted by physical activity, one modality of the PREVENTION intervention. We did not see an effect in the hippocampus, possibly due to small sample size. We did not find an effect of treatment arm, potentially because both receive recommendations and medical management, and did not differ in adherence.